# A Systematic Review and Meta‐Analysis of the Epidemiology of Bovine Herpesvirus 1 Infection in Dairy Cattle in Ethiopia

**DOI:** 10.1002/vms3.70908

**Published:** 2026-03-30

**Authors:** Tsegaye Asredie Kolech, Eyasu Demsie Molla, Dejen Asaye Mengistu

**Affiliations:** ^1^ Gondar Agricultural Research Center Gondar Ethiopia; ^2^ Gondar Zuria District Livestock Development Office Gondar Ethiopia; ^3^ Department of Veterinary Clinical Medicine School of Veterinary Medicine Bahir Dar University Bahir Dar Ethiopia

**Keywords:** bovine herpes virus 1, dairy cattle, Ethiopia, prevalence, systematic review and meta‐analysis

## Abstract

Bovine herpesvirus 1 (BoHV‐1) is a widely prevalent and economically important pathogen of cattle with substantial implications for dairy production in Ethiopia. This systematic review and meta‐analysis aimed to estimate the pooled prevalence of BoHV‐1 infection in the country. A systematic literature searches of PubMed, ScienceDirect, Scopus, and Google Scholar was conducted to identify eligible studies reporting the seroprevalence of bovine herpesvirus‐1 in dairy cattle. Pooled prevalence estimates were generated using a random‐effect meta‐analysis model. A total of nine studies that met the inclusion criteria were analysed, encompassing 5078 dairy cattle tested using competitive (c‐ELISA) and blocking (b‐ELISA) enzyme‐linked immunosorbent assays. The overall pooled prevalence of BoHV‐1 was estimated at 46.02% (95% CI: 33.36 to 58.68). A higher prevalence was observed with the c‐ELISA (54.17%; 95% CI: 31.62 to 76.71) compared to the b‐ELISA (39.56%; 95% CI: 26.08 to 53.04). Temporal analysis revealed a pooled prevalence of 36.93% (95% CI: 25.93 to 47.93 from studies conducted between 2018 and 2021 (3 studies, 2274 samples) and 56.63% (95% CI: 32.96 to 68.31) from studies between 2022 and 2025 (6 studies, 2804 samples). Prevalence also varied with sample size, with the highest prevalence recorded in studies with a sample size between 413–563 (51.79%; 95% CI: 41.37 to 62.21), followed by those with a sample size of 164–384 (46%; 95% CI: 18.84 to 75.05). The finding suggests a rising trend in BoHV‐1 infection, with no evidence of publication bias. In‐depth epidemiological studies and the development of effective prevention and control strategies to mitigate the effects of BoHV‐1 infection in Ethiopia.

## Graphical abstract

**This systematic review and meta‐analysis summarise the epidemiology of bovine herpesvirus‐1** (BoHV‐1) infection in dairy cattle in Ethiopia. Analysis of 9 studies involving 5078 cattle revealed a high pooled seroprevalence of 46.02%, with higher estimates from c‐ELISA and an increasing temporal trend from 2018 to 2025.

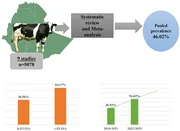

## 1 Introduction

Bovine herpes virus 1(BoHV‐1) belongs to the Herpesviridae family, order Herpesvirales, genus Varicellovirus, and subfamily Alphaherpesvirinae (Muylkens et al. 2007). BoHV‐1 is called the infectious bovine rhinotracheitis (IBR) virus of the alphaherpes virus that causes respiratory disease, abortion, and conjunctivitis (Constable et al. 2016). Genetically, at least three distinct subtypes of BoHV‐1, such as respiratory type (BoHV‐1.1), genital type (BoHV‐1.2), and encephalitic type (BoHV‐1.3) (Constable et al. 2016; Graham 2013; Metzler et al. 1985; Muylkens et al. 2007). BoHV‐1 is the causative agent of infectious bovine rhinotracheitis (IBR), infectious pustular vulvovaginitis (IPV), and infectious pustular balanoposthitis (IPB), and is also an important viral pathogen of cattle, found worldwide (Nandi et al. 2011; Waldeck et al. 2021).

The introduction and spread of BoHV‐1 mainly occur through direct contact between susceptible and infected cattle (Waldeck et al. 2021) through respiratory, vaginal, and ocular secretions (Muylkens et al. 2007). The virus causes severe clinical syndromes such as respiratory disease, genital disease, abortion, neurological disease, and systemic disease in cattle (Chen et al. 2018). In cattle, BoHV‐1 was reported for the first time in California, USA, in 1953 (Chen et al. 2018; Durham 1974; Raaperi et al. 2014; Tikoo et al. 1995). In the present time, the distribution of BoHV‐1 is worldwide (Hamdy et al. 2022). However, it has been eradicated in some European countries, such as Australia, Denmark, Finland, Sweden, Germany, and the Czech Republic (Mazzeo et al. 2024).

In Ethiopia, reports of BoHV‐1 infection have increased on commercial and smallholder dairy farms due to urbanisation and the intensification of dairy production over the last few decades. Understanding the epidemiology and prevalence of BoHV‐1 infection is essential for effective prevention and control strategies. To date, no systematic review and meta‐analysis have been conducted on the prevalence of BoHV‐1 infection in Ethiopia. Therefore, this study aims to systematically review and quantitatively analyse the prevalence and associated risk factors of BoHV‐1 infection in the country.

## 2 Materials and Methods

### 2.1 Searching Strategy

A systematic literature search was performed in April 2025 across PubMed, ScienceDirect, Scopus, and Google Scholar to identify Studies reporting BoHV‐1 infection in dairy cattle in Ethiopia. The search strategy was specifically designed to retrieve the seroprevalence data and followed a structured, targeted screening process.

PubMed searching strategy. Based on the MeSH terminologies, the following keywords were used to search: ‘herpes virus’, ‘infectious rhinotracheitis’, ‘cattle’, and the Boolean operators ‘OR’ and ‘AND’ in the keyword/title/summary field. (((((((((((((((((((((((((((prevalence OR (seroprevalence)) OR (Sero‐epidemiology)) AND (Viral bovine rhinotracheitis)) OR (IBR)) OR (Infectious bovine rhinotracheitis)) OR (infectious pustular vulvovaginitis)) OR (IPV)) OR (Red nose)) OR (Necrotic rhinitis)) OR (Bovine herpesvirus 1)) OR (Bovine herpesvirus ‐1)) OR (Bovine alphaherpesvirus 1)) OR (Bovine alphaherpesvirus ‐1)) OR (Bovine herpesvirus type 1)) OR (bovine alphaherpesvirus type 1)) OR (BoHV‐1)) OR (BoAHV‐1)) OR (BHV‐1)) OR (BoHV1)) OR (BoAHV1)) OR (BHV1)) AND (Cattle)) OR (Bovine)) OR (dairy cattle)) OR (dairy cows)) OR (dairy herd)) OR (dairy farms)) OR (heifers)

To enhance the accuracy of our results, we employed advanced search strategies across Scopus, ScienceDirect, and Google Scholar. Keywords such as ‘cattle’, ‘herpes virus’, ‘rhinotracheitis virus’, and ‘prevalence’ were used to identify and select relevant studies.

### 2.2 Inclusion and Exclusion Criteria

Eligible articles were screened based on predefined inclusion and exclusion criteria. Studies were included if they investigated the prevalence of BoHV‐1 infection, focused on dairy cattle, covered any production systems or study designs, were original research articles, and were published in English.

Exclusion criteria included duplicate articles, inaccessible full texts, articles that cannot be downloaded, studies involving vaccinated or experimental animals, unclear or incomplete data, sample size fewer than 30, studies on non‐cattle species (e.g., pigs, sheep, goats, camels, or wild ruminants), review articles, and publications not in English

### 2.3 Data Extraction

After removing all duplicates, citations identified through the search strategy were exported into EndNote version 7 for reference management. Two independent reviewers screened the titles and abstracts of the retrieved studies, excluding those that did not meet the eligibility criteria. Full‐text articles of the selected studies were then obtained and thoroughly reviewed to confirm their inclusion. Additionally, a manual search of the reference lists of selected articles was conducted to identify any relevant studies that may have been missed during the initial search.

Data were extracted independently by two reviewers using a standard data collection form designed to capture key variables relevant to research objectives. An impartial third author resolved disagreements between the two authors regarding data extraction. The data extraction included primary authorship, publication year, sample size, prevalence rate, study design, and study location. Each selected study was reviewed multiple times, and both qualitative and quantitative data were extracted using a pre‐established data extraction template.

### 2.4 Quality Assessment

The methodological quality of the included studies was evaluated using the Joanna Briggs Institute (JBI) critical appraisal checklist for prevalence studies (Munn et al. 2015). Three reviewers independently assessed each study using the eight‐item checklist designed for cross‐sectional studies. Each item was scored as 1 for ‘yes’ and 0 for ‘no’, with total scores summarised in Table 1. Any discrepancies in scoring between reviewers were examined and resolved through discussion and consensus.

**TABLE 1:** Quality assessment of all studies included in the systematic review was evaluated using the JBI critical appraisal tool.

Author(s)	Were the inclusion criteria for the sample clearly defined?	Were the study subjects and the setting described in detail?	Was the exposure measured validly and reliably?	Were objective, standard criteria used for the measurement of the condition?	Were confounding factors identified?	Were strategies to deal with confounding factors stated?	Were the outcomes measured validly and reliably?	Was an appropriate statistical analysis used?	Score out of 8
Kolech et al. (2025)	Yes	Yes	Yes	Yes	Yes	Yes	Yes	Yes	8
Engdawork et al. (2024)	Yes	Yes	Yes	Yes	No	No	Yes	Yes	6
Girmay et al. (2024)	Yes	Yes	Yes	Yes	No	No	Yes	Yes	6
Danu et al. (2024)	Yes	Yes	Yes	Yes	No	No	Yes	Yes	6
Asmare et al. (2023)	Yes	Yes	Yes	Yes	Yes	Yes	Yes	Yes	8
Shewie et al. (2023)	Yes	Yes	Yes	Yes	No	No	Yes	Yes	6
Tadeg et al. (2021)	Yes	Yes	Yes	Yes	No	No	Yes	Yes	6
Asmare et al. (2018)	Yes	Yes	Yes	Yes	No	No	Yes	Yes	6
Sibhat et al. (2018)	Yes	Yes	Yes	Yes	Yes	Yes	Yes	Yes	8

### 2.5 Statistical Analysis

Data analysis was performed using Stata version 17.0 (Stata Corp). Descriptive statistics were used to summarise data extracted from included studies and presented in the evidence tables. A meta‐analysis was conducted to estimate the pooled prevalence of BoHV‐1, using the Stata meta command to analyse proportions as the effect measure. To explore potential source heterogeneity, meta‐regression and subgroup analysis were performed. Between‐study heterogeneity was evaluated using the *I* ^2^ statistic and Cochrane's *Q* test (reported as χ^2^ and *p*‐values) under a random effect model. A forest plot was generated to display the individual and pooled prevalence estimates of BoHV‐1, along with 95% confidence intervals, study authors, and publication year. A funnel plot was used to explore potential publication bias. A meta‐analysis was performed according to the PRISMA guideline and checklists (Shamseer et al. 2015).

## 3 Results

### 3.1 Characteristics Included in Studies

**FIGURE 1:**
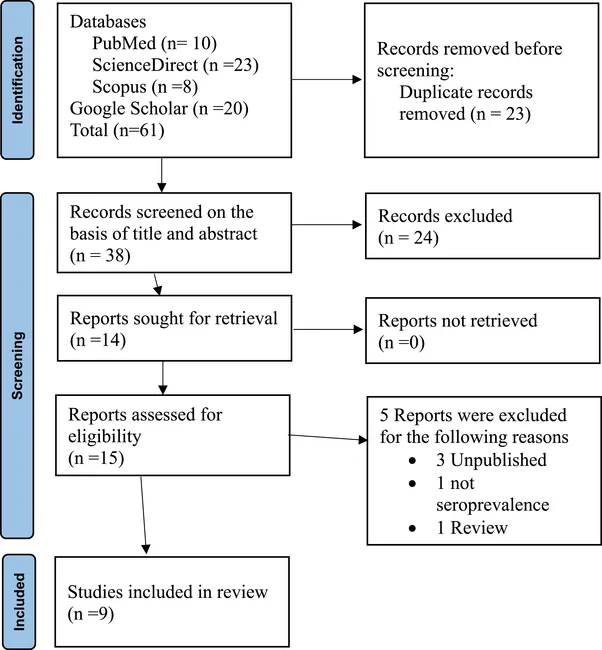
Flow diagram illustrating the identification, screening, eligibility assessment, and final inclusion of studies in the present systematic review.

A total of 61 records were identified through the systematic literature search (Figure 1). After removing 23 duplicate records, 38 unique studies underwent title and abstract screening. Of these, 5 studies were excluded based on the predefined inclusion and exclusion criteria.

### 3.2 Included Studies

In this literature search, a total of nine studies were included in the meta‐analysis of BoHV‐1 (Table 2), with the majority conducted in the central part of Ethiopia.

**TABLE 2:** Summary of the key characteristics of each study included in the systematic review and meta‐analysis of bovine herpes virus‐1 (BoHV‐1).

Author	Location	Study design	ELISA type	Sample size	No. of positive animals	Prevalence (%)
Kolech et al. (2025)	Northwest	CS	c‐ELISA	431	214	49.65
Engdawork et al. (2024)	Central	CS	b‐ELISA	511	316	61.84
Girmay et al. (2024)	Central, North	CS	c‐ELISA	164	100	60.98
Danu and Deresa (2024)	West	CS	c‐ELISA	384	309	80.47
Asmare et al. (2023)	Southern and central	CS	b‐ELISA	945	286	30.26
Shewie et al. (2023)	Addis Ababa	CS	b‐ELISA	369	77	20.87
Tadeg et al. (2021)	Northeast	CS	c‐ELISA	332	85	25.60
Asmare et al. (2018)	Central, south, and south‐western	CC	b‐ELISA	563	247	43.87
Sibhat et al. (2018)	Central, southern, and southwestern	CS	b‐ELISA	1379	566	41.04

CS, cross‐sectional; CC, case‐control.

### 3.3 Pooled Prevalence of BoHV‐1

**FIGURE 2:**
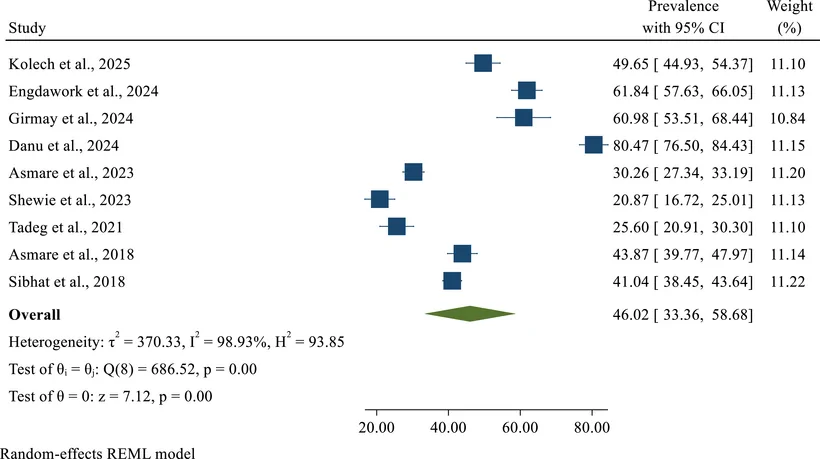
Pooled seroprevalence of bovine Herpes virus‐1 (BoHV‐1) in dairy cattle, with 95% confidence intervals, estimated using a random effect model based on included studies.

Using a random‐effects model, the pooled prevalence of BoHV‐1 in dairy cows was estimated at 46.02% (95% CI: 33.36 to 58.68) (Figure 2). There was substantial heterogeneity among the included studies (*I* ^2^ = 98.93%; *p* < 0.000).

### 3.4 Subgroup Analysis

We performed Subgroup analysis to identify potential sources of heterogeneity by publication year, diagnostic technique, and sample size (Table 3). The result showed that BoHV‐1 prevalence varied notably across subgroups. The pooled prevalence was higher with the competitive ELISA (c‐ELISA), 54.17% (95% CI: 31.62 to 76.71), than with the blocking ELISA (b‐ELISA), 39.56% (95% CI: 26.08 to 53.04). In terms of publication year, three studies conducted between 2018 and 2021 tested 2274 samples, yielding a pooled prevalence of 36.93% (95% CI: 25.93 to 47.93). In contrast, six studies from 2022 to 2025 analysed 2804 samples and reported a higher pooled prevalence of 56.63% (95% CI: 32.96 to 68.31), indicating a rising trend over time. When subgroup analysis by sample size, the highest pooled prevalence was observed in studies with sample sizes ranging from 413 to 563, with a pooled prevalence of 51.79% (95% CI: 41.37 to 62.21). The second‐highest prevalence, 46% (95% CI: 18.84 to 75.05), was observed in studies with a sample size of 164–384.

**TABLE 3:** Subgroup analysis of bovine herpesvirus‐1(BoHV‐1) seroprevalence in dairy cattle by ELSA type, publication year, and sample size.

Variable	Category	No. studies	No. examined	No. positive	Prevalence (%)	Heterogeneity			
						95% CI	χ^2^	*p*‐value	*I* ^2^ (%)
ELISA type	b‐ELISA	5	3767	1492	39.56	(26.08 to 53.04)	228.08	0.000	98.68
	c‐ELISA	4	1311	708	54.17	(31.62 to 76.71)	315	0.000	98.79
Publication year	2018–2021	3	2274	898	36.93	(25.93 to 47.93)	39.09	0.000	90.59
	2022–2025	6	2804	1302	56.63	(32.96 to 68.31)	613.86	0.000	99.06
Sample size	164–384	4	1249	2038	46.95	(18.84 to 75.05)	519.3	0.000	99.28
	413–563	3	1505	1895	51.79	(41.37 to 62.21)	36.99	0.000	94.23
	945–1379	2	2324	852	35.68	(25.11 to 46.24)	29.14	0.000	96.57
Total		9	5078	2200	46.02	(33.36 to 58.68)	686.52	0.000	98.93

### 3.5 Meta‐Regression

A univariate meta‐regression analysis was performed to identify a further potential source of heterogeneity. Among the evaluated risk factors for BoHV‐1 infection, a history of abortion was found to be significantly associated with BoHV‐1 infection (*p* < 0.05) (Table 4). In contrast, the other risk factors examined in the individual studies did not show a significant association with BoHV‐1 infection in this meta‐analysis.

**TABLE 4:** Meta‐regression analysis showing factors contributing to the heterogeneity and estimated pooled prevalence of BoHV‐1 in dairy cattle.

Factor	Category	No. of studies	No. examined	No. positive	Prevalence (95% CI)	Univariate meta‐regression	
						Coefficient (95% CI)	*p*‐value
Breed	Local	2	455	289	62.84 (56.50 to 69.18)	Ref.	
	Cross	2	440	344	70.93 (36.59 to 105.27)	10.07 (25.47 to 45.61)	0.579
Age	Young	5	685	221	32.53 (8.23 to 56.83)	Ref.	
	Adult	5	1891	984	57.04 (38.77 to 75.31)	24.55 (–5.82 to 54.92)	0.113
Parity	Nulliparous	3	312	95	24.63 (0.94 to 48.33)	Ref.	
	Uniparous	4	469	168	36.11 (12.61 to 59.61)	11.42 (–23.73 to 46.58)	0.524
	Multiparous	4	760	358	50.18 (26.19 to 74.17)	25.50 (–9.55 to 60.55)	0.154
Breeding method	Bull	4	462	251	42.79 (8.76 to 76.82)	Ref.	
	AI	4	585	154	51.14 (6.14 to 96.15)	−0.11 (–0.73 to 0.51)	0.727
	Both	3	719	252	59.33 (26.77 to 91.90)	0.002 (–0.65 to 0.66)	0.994
Herd size	Small	3	342	189	49.89 (3.40 to 96.38)	Ref.	
	Medium	3	329	134	66.31 (21.94 to 110.69)	16.43 (–45.37 to 78.24)	0.602
	Large	2	64	32	65.54 (21.22 to 109.87)	15.91 (–54.03 to 85.85)	0.656
History of abortion	No	3	896	248	28.85 (–3.01 to 60.72)	Ref.	
	Yes	3	106	80	82.52 (63.71 to 101.34)	53.60 (16.11 to 91.10	0.005
Retained fetal membrane	No	3	800	443	29.46 (–0.92 to 59.85)	Ref.	
	Yes	3	202	114	58.41 (34.09 to 82.73)	28.10 (–10.10 to 68.14)	0.147
Repeat breeder	No	2	421	57	13.54 (10.27 to 16.80)	Ref.	
	Yes	2	280	105	42.37 (13.02 to 71.71)	28.51 (0.05 to 56.97)	0.050

Ref, reference; CI, confidence interval.

### 3.6 Assessment of Publication Bias

**FIGURE 3:**
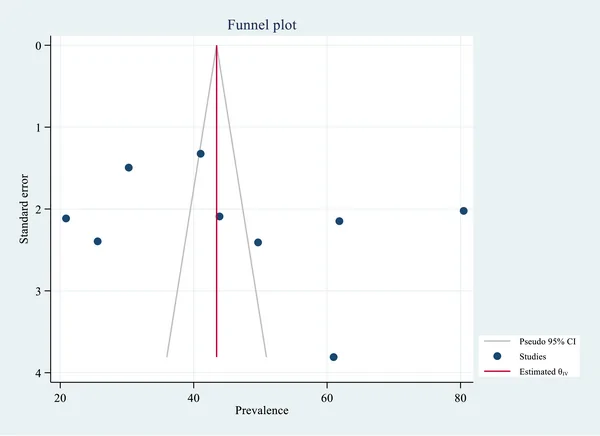
Funnel plot assessing potential publication bias among the included studies, showing individual study effect sizes plotted against their standard errors, with pseudo 95% confidence limit around the pooled prevalence estimate.

Publication bias in the selected studies was evaluated using a funnel plot (Figure 3). Visual inspection of the plot revealed no marked asymmetry, which was confirmed by Egger's regression test (β = 0.00, SE = 1.30, *p* = 1.00), indicating no evidence of publication bias.

## 4 Discussion

Based on nine eligible studies, this work represents the first systematic review and meta‐analysis study assessing bovine herpesvirus‐1 (BoHV‐1) infection in dairy cattle in Ethiopia. The pooled prevalence of BoHV‐1 in Ethiopia was estimated at 46.02%, indicating a substantial burden of the virus among dairy cattle. Regionally, the highest seroprevalence was observed in Western Ethiopia (80.47%), followed by Central Ethiopia (61.84%), while the lowest was recorded in Addis Ababa (20.87%). This finding suggests that BoHV‐1 is widely distributed across the country. BoHV‐1 infection in dairy cattle indicates that the virus was widespread throughout Ethiopia. The findings of this systematic review and meta‐analysis indicate an increasing trend in the seroprevalence of BoHV‐1 infection, underscoring its growing importance as a health and economic concern in the Ethiopian dairy sector.

The current pooled prevalence of BoHV‐1 in Ethiopia was 46.02% higher than the 40% reported in a systematic review and meta‐analysis by Chen et al. (2018) in China. In contrast, it was lower than the 57.76% pooled prevalence observed by Demeli and Fındık (2022) in Turkey. The higher prevalence of BoHV‐1 might be associated with a high level of contact among individual animals within a large herd and intensively farmed animals (Snowder et al. 2006). BoHV‐1 prevalence differences among countries may be attributed to variations in production systems, breeding methods, herd size, animal age, and differences in disease control measures (Ackermann and Engels 2006; Kipyego et al. 2020). The prevalence differences are also due to the specificity and sensitivity of the diagnostic tests for BoHV‐1(Graham et al. 1998). In addition, the accuracy of diagnostic tests has become a common method for the detection of BoHV‐1 infection, as they are time‐efficient, cost‐effective, rapid, and require minimal specialised equipment and staff training (Chen et al. 2018).

The virus neutralisation test (VNT) is the gold standard test of the World Organization for Animal Health (OIE) for the laboratory diagnosis of BoHV‐1 infection. In addition, the enzyme‐linked immunosorbent assay (ELISA) is also approved for international trade (Godhardt‐Cooper et al. 2009). The prevalence of BoHV‐1 detected using blocking ELISA (39.56%) was lower than that detected using competitive ELISA (54.17%). This difference may be attributed to the characteristics of blocking ELISA, which exhibits high sensitivity, low false‐positive rates, and minimal variability when applied to undiluted bovine serum (Aydin 2015; Kramps et al. 1994). described that blocking ELISA demonstrates very high sensitivity compared with competitive ELISA.

The systematic review and meta‐analysis results revealed a significant difference in the prevalence of BoHV‐1 infection between the 2018–2021 period (36.93%) and the 2022–2025 period (56.63%), indicating that the condition of BoHV‐1 infection in Ethiopia has worsened and become more severe in recent years. This result is consistent with the study of Chen et al. (2018) in China. This might be due to the extensive laboratory investigations and continuous monitoring of BoHV‐1 infection and its associated risk factors, which support recommendations designed to reduce the disease incidence.

The prevalence of BoHV‐1 infection in some European countries may be influenced by various factors, including herd size and type, the presence of bulls, breed, sex, age, cattle purchasing practices, communal grazing, the distance between herds, and the presence of visitors (Dias et al. (2013); Waldeck et al. 2021). reported that BoHV‐1 infection was associated with factors including herd type, purchasing of breeding cattle, natural service, rental of grazing areas, presence of a calving pen, and a history of abortion in southern Brazil. In most studies included in this systematic review and meta‐analysis, the commonly reported risk factors for BoHV‐1 infection were age group, breeding type, parity, breed, herd size, history of abortion, retained fetal membranes, and repeat breeding. However, further analysis revealed that only the history of abortion was significantly associated with BoHV‐1 infection (*p* < 0.05). The other identified risk factors did not show a statistically significant association with BoHV‐1 infection. Additionally, some of the eligible studies included in this meta‐analysis lacked sufficient data on risk factors.

## 5 Conclusions

This meta‐analysis revealed that BoHV‐1 infection is widespread in Ethiopia, with particularly high prevalence in the central regions. BoHV‐1 has remained a significant pathogen affecting dairy cows in the country for decades. The pooled prevalence varied based on ELISA type, publication year, and sample size. To better understand and control the disease, further large‐scale, comprehensive studies are recommended, along with the development of effective prevention and control strategies.

## Author Contributions

**TAK**: conceptualisation, data curation, formal analysis, methodology, software, supervision, validation, visualisation, writing – original draft, writing – review, and editing. **EDM**: conceptualisation, formal analysis, methodology, supervision, visualisation, writing – original draft, writing – review, and editing. **DAM**: conceptualisation, data curation, formal analysis, methodology, supervision, validation, visualisation, writing – original draft, writing – review, and editing.

## Funding

The authors have nothing to report.

## Ethics Statement

The authors have nothing to report.

## Conflicts of Interest

The authors declare that no conflict of interest.

## Data Availability

The data that were extracted and analysed for this study are available from the corresponding author upon request.
